# Allelic Dropout During Polymerase Chain Reaction due to G-Quadruplex Structures and DNA Methylation Is Widespread at Imprinted Human Loci

**DOI:** 10.1534/g3.116.038687

**Published:** 2017-01-30

**Authors:** Aaron J. Stevens, Millie G. Taylor, Frederick Grant Pearce, Martin A. Kennedy

**Affiliations:** * Department of Pathology, University of Otago, Christchurch 8140, New Zealand; † Biomolecular Interaction Centre, School of Biological Sciences, University of Canterbury, Christchurch 8140, New Zealand

**Keywords:** G-quadruplex, PCR, allelic dropout, methylation, polymerase chain reaction

## Abstract

Loss of one allele during polymerase chain reaction (PCR) amplification of DNA, known as allelic dropout, can be caused by a variety of mechanisms. Allelic dropout during PCR may have profound implications for molecular diagnostic and research procedures that depend on PCR and assume biallelic amplification has occurred. Complete allelic dropout due to the combined effects of cytosine methylation and G-quadruplex formation was previously described for a differentially methylated region of the human imprinted gene, *MEST*. We now demonstrate that this parent-of-origin specific allelic dropout can potentially occur at several other genomic regions that display genomic imprinting and have propensity for G-quadruplex formation, including *AIM1*, *BLCAP*, *DNMT1*, *PLAGL1*, *KCNQ1*, and *GRB10*. These findings demonstrate that systematic allelic dropout during PCR is a general phenomenon for regions of the genome where differential allelic methylation and G-quadruplex motifs coincide, and suggest that great care must be taken to ensure biallelic amplification is occurring in such situations.

DNA amplification by polymerase chain reaction (PCR) is an enzymatic technique for the *in vitro* synthesis of targeted DNA regions, mediated by thermally stable DNA polymerases. PCR is a prerequisite for most experimental procedures that involve DNA detection, sequencing, cloning, and genotyping (Mullis and Faloona 1987; Saiki *et al.* 1988; Bevan *et al.* 1992), and is of widespread application in genetic research and molecular diagnostic applications (Desforges and Eisenstein 1990). Despite extensive optimization and near ubiquitous usage, PCR is still prone to failure under certain circumstances. For diploid organisms, the failure of one allele to amplify can result in allelic dropout (ADO), causing apparent homozygosity (Askree *et al.* 2011; Boán *et al.* 2004; Lam and Mak 2013; Landsverk *et al.* 2012; Piyamongkol *et al.* 2003; Saunders *et al.* 2010; Wenzel *et al.* 2009). ADO is an insidious problem that is difficult to recognize because the PCR appears successful, but half of the genetic information is missing. ADO can have significant implications in both research and clinical applications, where there is a requirement for high sensitivity and accurate PCR genotyping. Incorrect genotyping can have substantial negative consequences and may result in the misdiagnosis of genetic diseases, loss of the ability to differentiate between individuals, and false assumptions about parentage or genetic diversity (Boán *et al.* 2004; Landsverk *et al.* 2012; Saunders *et al.* 2010; Tomaz *et al.* 2010; Wenzel *et al.* 2009).

We previously characterized a novel mechanism of ADO that occurred during genotyping of the imprinted human gene, *MEST* (Stevens *et al.* 2014). Extensive PCR analysis of a short region in the *MEST* promoter invariably led to non-Mendelian genotype patterns for three single nucleotide polymorphisms (SNPs), which could not be resolved by primer redesign or standard PCR optimization strategies. We established that both cytosine methylation and DNA structures known as G-quadruplexes (G4s) in this region contributed to ADO, leading to incorrect genotyping (Stevens *et al.* 2014). G4 are secondary DNA structures that can form in G-rich regions due to the self-association of guanine through Hoogsteen bonds. Four guanine nucleotides can adopt a square planar arrangement, referred to as a G-quartet, and multiple G-quartets can then stack upon one another to form a G4 (Sen and Gilbert 1988; Sundquist and Klug 1989). G4 formation is stabilized by the integration of a cation, like potassium (Ambrus *et al.* 2005; Biffi *et al.* 2012; Maizels 2015; Rhodes and Lipps 2015; Simonsson 2001), making PCR buffer an optimal environment for G4 formation. G4 structures may then act as a steric block to Taq polymerase (Boán *et al.* 2004; Chambers *et al.* 2015; Saunders *et al.* 2010; Weitzmann *et al.* 1996), an effect that is exacerbated when the G4 region is methylated (Stevens *et al.* 2014).

During amplification of the imprinted *MEST* promoter region, we observed consistent ADO of the maternally inherited, methylated allele. Correct genotypes from this locus were only obtained using extraordinary modifications of PCR, including methylation-specific PCR, allele-specific enzymatic digestion of genomic DNA, and PCR buffers lacking potassium. We demonstrated that ADO resulted from the combination of both cytosine methylation and guanine Hoogsteen bonds in the template DNA, which can form G4 structures, and that neither factor in isolation was sufficient to cause complete ADO (Stevens *et al.* 2014).

The novel form of ADO observed at the *MEST* promoter region was intriguing and problematic, but it was unclear if it was a phenomenon restricted solely to this genomic region or a more general occurrence throughout the genome. In this report, we describe the design of an assay and its application to test for the potential occurrence of ADO during amplification of differentially methylated DNA. The templates used in this assay were generated by PCR from a range of imprinted genes, and *in vitro* methylation with the enzyme M.SssI was used to mimic differentially methylated alleles. After demonstrating potential ADO using the assay on multiple synthetic model templates, we then demonstrated that this type of ADO could be observed in genomic DNA analysis of an imprinted human locus other than *MEST*.

## Materials and Methods

### Selection of G4s for analysis

Imprinted genes with a confirmed parent-of-origin methylation status (Jirtle 2012; Morison *et al.* 2001) were analyzed for G4-forming motifs using the bioinformatic software, QGRS Mapper (Kikin 2006). G4 motifs that contained runs of three or more guanines and a loop length between 0 and 7 nt were considered for analysis, as these were most likely to adopt G4 structure. This corresponded with a QGRS mapper score of at least 37, which was therefore selected as an arbitrary threshold for G4 cutoff. PCR amplicons of ∼300 bp were designed from these regions, to contain a single G4 motif and a CCGG endonuclease recognition sequence for *Hpa*II and *Msp*I endonucleases (Supplemental Material, Figure S1 and Table S1 in File S1).

### PCR

PCR was carried out in a Mastercycler pro thermal cycler (Eppendorf, Stevenage, UK) with Fisher Taq-ti polymerase (Fisher Biotec, WA, Australia). The initial denaturation step consisted of 95° for 2 min, and extension was performed at 72°. Cycling conditions consisted of denaturation at 95° for 15 sec, annealing for 15 sec, and extension for 45 sec. The initial annealing temperature was 65°, and this was decreased by 1° per cycle for 10 cycles, followed by 25 cycles at 55°. A final extension was performed for 5 min.

### Sanger DNA sequencing

PCR products were prepared for Sanger DNA sequencing by purification using AcroPrep (PALL Corporation, Port Washington, NY) 96-well filter plates (Omega 30K). Purified PCR amplicons were then resuspended in water and ∼10 ng was sequenced with the appropriate primer, using BigDye Terminator v3.1 Cycle Sequencing Kit (Applied Biosystems, Foster City, CA), following the manufacturer’s protocol. Sequencing reaction products were run on an AB3130xl fragment analysis system equipped with a 50-cm capillary, using POP7 polymer.

### Synthetic DNA templates

Selected G4-containing amplicons (Figure S1 in File S1) were amplified by PCR, and as described below (*ADO assay on synthetic templates*), a single nucleotide difference was introduced to allow alleles to be distinguished. *In vitro* methylation and digestion experiments were performed on PCR products generated from genomic DNA, using enzymes purchased from New England Biolabs Inc., (Ipswich, MA). *In vitro* methylation was carried out by incubation with M.SssI for 120 min at 37°, followed by heat inactivation at 65° for 20 min, as recommended by the manufacturer’s protocol. To assess the extent of *in vitro* methylation achieved, each methylated DNA template was then incubated with the restriction enzyme *Msp*I and its methylation-sensitive isoschizomer *Hpa*II. The resulting digestion products were analyzed by gel electrophoresis and compared, to ensure successful methylation (data not shown).

### ADO assay on synthetic templates

**Figure 1:**
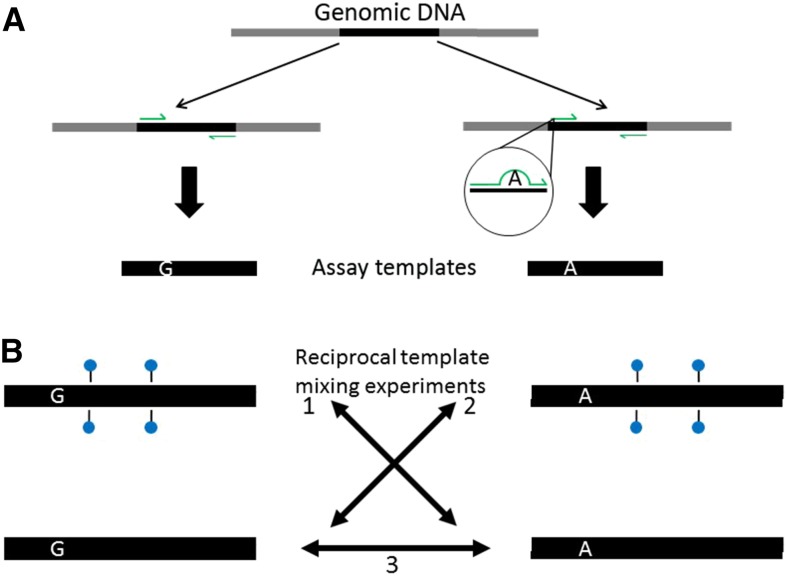
Synthetic template ADO assay (A) Two templates (black bars) were generated by targeted PCR amplification of genomic DNA (gray). These assay templates differed by a single base pair, which was introduced into one template (right) through primer-directed mutagenesis (small green arrows and enlarged view). (B) An aliquot of each template was then methylated (blue circles) with M.SssI, and reciprocal mixing experiments were performed on each template combination as shown. In total, three template mixing experiments were performed per amplicon, for example: (1) methylated G *vs.* nonmethylated A; (2) methylated A *vs.* nonmethylated G; and (3) nonmethylated A *vs.* nonmethylated G.

The assay for modeling methylation specific ADO was based on the method described by Stevens *et al.* (2014). Synthetic DNA templates generated by PCR of genomic DNA were used for this assay because the endogenous regions did not contain the necessary SNPs for detecting ADO. During PCR, an artificial SNP was introduced by primer mutagenesis (Simsek and Adnan 2000) to create two alleles that which could be distinguished by Sanger sequencing (Figure 1A and Figure S1 and Figure S2 in File S1). This meant that for each amplicon there was a wild type and mutant template that differed by a single base pair near the 3′ end of the forward primer (Figure S2 in File S1). Aliquots of each synthetic template were then subjected to in vitro methylation with the CpG methyltransferase M.SssI (as described in *Synthetic DNA templates*), to produce four different template types (Fig. 1B). For example, if the artificial SNP alleles were either an A or G, the different possible combinations were A methylated, A non-methylated, G methylated, and G non-methylated.

Methylated and nonmethylated amplicons were mixed in various combinations and used to seed PCR, the products of which were then genotyped by Sanger sequencing. For each region, three different mixing experiments were performed, two consisting of reciprocal pairs of methylated and nonmethylated templates (mimicking maternal or paternal alleles for imprinted genomic DNA), and one consisting of both nonmethylated templates (Figure 1B). Mixing of the nonmethylated templates was a control to ensure that introducing an artificial SNP into the DNA sequence did not influence the genotyping outcome. Each assay was repeated at least two times, for each gene region.

### ADO during genomic DNA analysis of PLAGL1

Genomic *PLAGL1* analysis was performed on DNA samples NA19312 and NA20588 (Coriell Institute for Medical Research, Camden, NJ) that were known to be heterozygous at SNP rs2281476. PCR amplification using low potassium PCR buffer (Stevens *et al.* 2014), followed by Sanger sequencing, was used to detect heterozygosity at SNP rs2281476. To demonstrate ADO, genotypes obtained in low potassium buffer were compared to genotypes obtained in standard PCR buffer (containing 50 mM KCl).

The methylation status of each allele in genomic DNA for the *PLAGL1* amplicon was interrogated using methylation-dependent digestion by McrBC and *Hpa*II endonucleases (New England Biolabs Inc.). This was performed on ∼70 ng genomic DNA according to the manufacturer’s protocol. PCR was then separately performed on the differentially digested genomic DNA aliquots and the amplicons were analyzed by Sanger sequencing. To determine the methylation status of the allele that failed to amplify, the genotyping results obtained in this way were compared to those derived from standard PCR performed on nondigested genomic DNA of known haplotypes.

### Circular dichroism spectroscopy

Oligonucleotides were purchased from Integrated DNA Technologies (IDT Pte. Ltd., Singapore) and assessed for G4 formation in 10 mM Tris-HCl, 1.5 mM MgCl_2_ (pH 7), in the presence and absence of 50 mM KCl. A total of 4 µM of oligonucleotide was heated at 95° for 10 min then cooled slowly to room temperature (22°) overnight. Circular dichroism (CD) measurements were performed on a J-815 CD Spectrometer (Jasco Analytical Instruments, Easton, MD), using a 1-mm path length quartz cuvette. Spectra were collected across 340–200 nm in 1-nm increments at both 25° and 95°. The reported spectra corresponded to the average of three scans, and an appropriate buffer blank was made for all spectra.

### Data availability

File S1 contains detailed descriptions of all DNA sequences, oligonucleotide sequences, and original CD spectroscopy data.

## Results and Discussion

### Modeling ADO with synthetic templates

We previously demonstrated that the colocalization of methylation with G4 structure results in ADO during PCR amplification of the human *MEST* promoter region. This is caused by both G4 formation and cytosine methylation in the DNA template, with neither factor alone being sufficient to cause ADO (Stevens *et al.* 2014). To determine the potential for more widespread occurrence of this phenomenon, we have now tested G4 motif–containing regions of several other imprinted genes. For this purpose, we used synthetic templates generated by PCR (with introduction of an artificial SNP to allow differentiation of alleles) followed by *in vitro* methylation. Mixing of these alleles in specific combinations allowed us to mimic monoallelic methylation of genomic DNA derived from several human imprinted genes. Eight regions from six genes (*AIM1*, *BLCAP*, *DNMT1*, *PLAGL1*, *KCNQ1*, and *GRB10*) were investigated using this ADO assay. For the genes *DNMT1* and *PLAGL1*, two separate amplicons were studied from the same gene, and the second amplicon is described as DNMT1 (B) or PLAGL1 (B) (Figure S1 and Table S1 in File S1).

**Figure 2:**
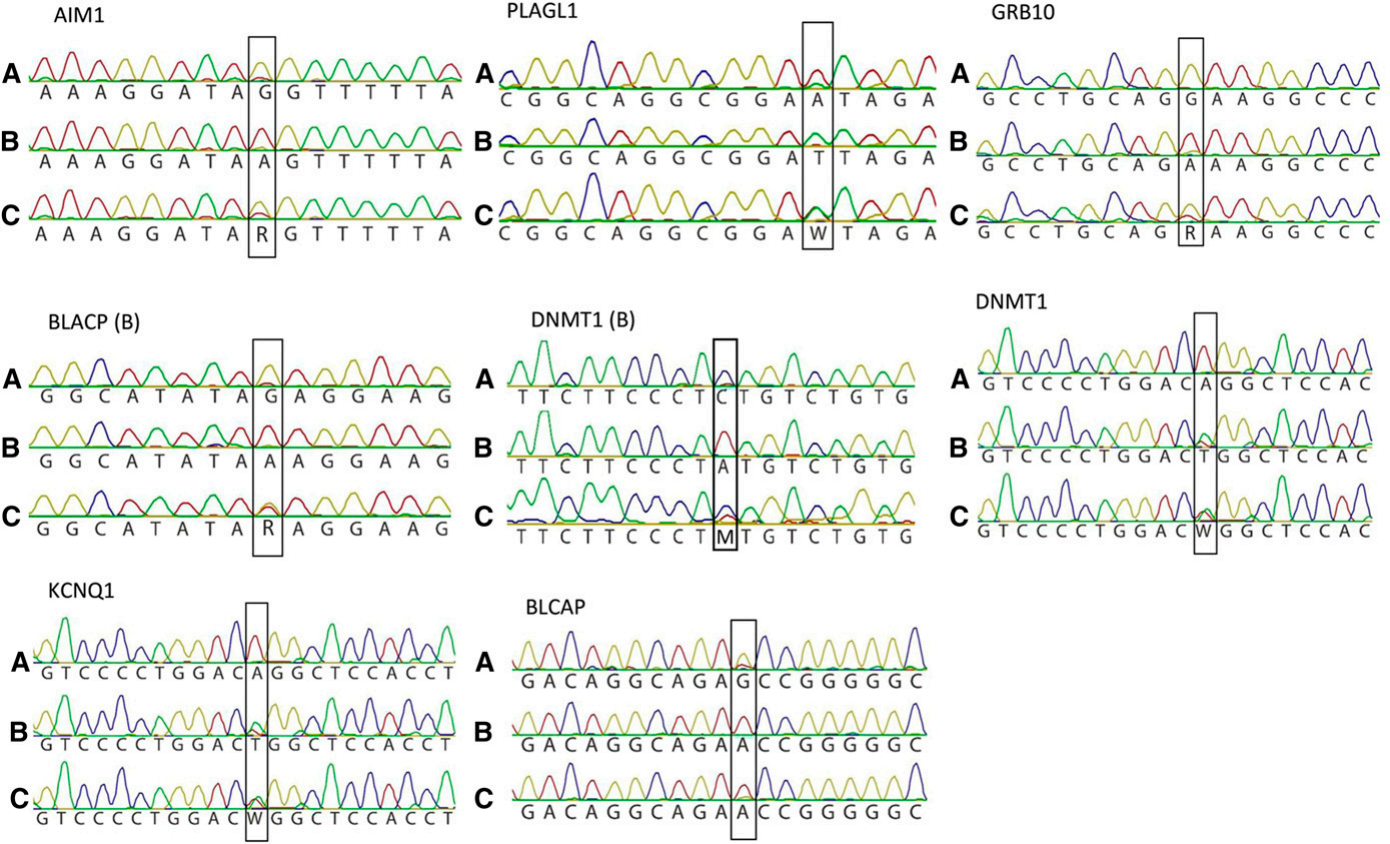
Sanger sequencing of PCR amplicons from synthetic template experiments. Sanger sequencing analysis of eight synthetic templates derived from imprinted gene regions. The black boxes indicate the position of the introduced artificial SNP. (A) Mix of methylated and nonmethylated templates. (B) Reciprocal methylated and nonmethylated template mixing experiment. (C) Mix of two nonmethylated templates.

Each of the eight assayed amplicons demonstrated ADO of the methylated template in at least one of the two reciprocal mixing experiments (Figure 2). Clear ADO of the methylated allele was always observed in mixing experiments with templates AIM1, PLAGL1, GRB10, BLCAP (B), and DNTM1 (B), with only the nonmethylated template detected during genotyping (by Sanger sequencing) (Figure 2). For these regions, amplification products from the mix of two nonmethylated templates gave heterozygous genotypes, with both alleles successfully detected in all cases (Figure 2).

Amplicons from KCNQ1 and DNMT1 demonstrated clear ADO in only one of the two methylated template–mixing experiments, with a minor peak from the methylated template visible in the reciprocal mixing experiment for each amplicon, indicating partial ADO (Figure 2). For these two amplicons, mixing of the nonmethylated templates demonstrated a clear heterozygous pattern in the Sanger sequencing traces (Figure 2). Because methylated and nonmethylated templates had identical sequences (except for the introduced artificial SNP), partial ADO during the reciprocal mixing experiment was likely to reflect inefficient methylation by M.SssI. Although the efficiency of methylation was assessed using restriction digest by *Hpa*II and *Msp*I, this can only detect methylated cytosine within the recognition sequence CCGG, and not at additional CpG dinucleotides present in each amplicon (Figure S1 in File S1).

Amplicons from BLCAP demonstrated ADO of each methylated template during the mixing experiments, however, when the nonmethylated templates were mixed, ADO was also apparent (Figure 2). This may indicate that the introduction of the artificial SNP decreased the amplification efficiency of the G template. However, there was still clear evidence of ADO when each template was methylated.

Many factors may potentially interact to direct ADO during PCR of differentially methylated regions that contain G4, and further research is required to completely understand this process. Amplicon size did not appear to correlate with the propensity for ADO, which predominantly appears to be determined by the position and stability of the G4. We did not determine if the number or position of methylated CpGs also contributed toward ADO.

The data presented here demonstrate that, for regions spanning a G4 motif, amplification of nonmethylated DNA is always favored during PCR, leading to ADO of the methylated allele. This confirmed that parent-of-origin specific ADO in regions of differential methylation that contain a G4 motif is not unique to *MEST*, and is a more general phenomenon likely to occur at many imprinted regions of the genome.

### ADO at the genomic PLAGL1 locus

Because the synthetic template assay is a model of differentially methylated DNA, we sought to extend our analysis to an endogenous region of imprinted genomic DNA other than the *MEST* promoter (Stevens *et al.* 2014), where this form of ADO was originally described. The genomic regions that were used to generate synthetic templates for the ADO assay did not contain common endogenous SNPs, which are necessary for the detection of ADO by Sanger sequencing. However, a region located ∼200 bp upstream of the PLAGL1 (B) amplicon contains a SNP (rs2281476) with a minor allele frequency of ∼25% in Europeans (Lek *et al.* 2016). This region is a differentially methylated CpG island associated with the promoter of *PLAGL1* (Yuen *et al.* 2011; Choufani *et al.* 2011), and rs2281476 is located within 130 bp of two G4 motifs that are situated on opposite DNA strands (Figure S3 in File S1). This combination of G4 motif, imprinted methylation, and presence of an SNP marked this region as a good target for detection of ADO.

**Figure 3:**
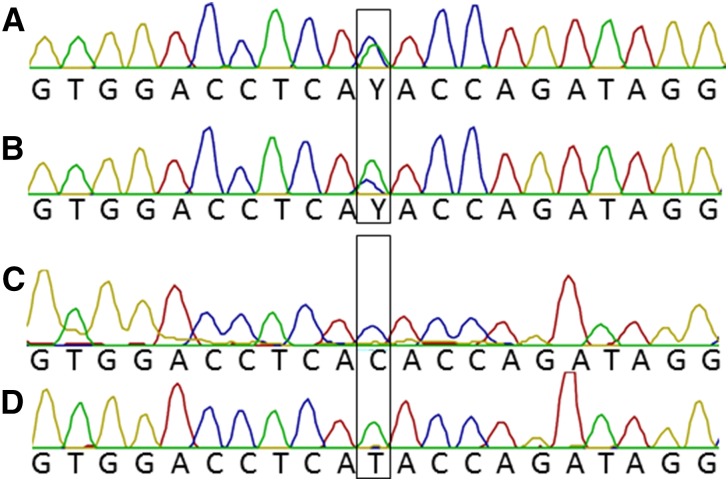
ADO analysis of genomic *PLAGL1*. Sanger sequencing results from PCR amplification of PLAGL1 amplicon (hg19, chr6:144328968-144328978) using primer PLAGL1Fa and PLAGL1Ra (Table S1 in File S1). The black boxes indicate the position of SNP rs2281476. Genotyping result obtained from PCR amplification in buffer lacking KCl for DNA sample NA20588 (A) and DNA NA19312 (B). Genotyping result obtained from PCR amplification in buffer containing 50 mM KCl for DNA sample NA20588 (C) and DNA sample NA19312 (D).

Genomic DNA samples were screened by PCR amplification using low potassium PCR buffer (to prevent G4 formation and ADO) (Stevens *et al.* 2014), followed by Sanger sequencing, to identify individuals heterozygous at rs2281476. Although several heterozygous individuals were identified, results were consistent and data from only two (DNA samples NA19312 and NA20588) are presented (Figure 3, A and B). Amplification of these genomic DNA samples using standard PCR buffer (50 mM KCl) revealed consistent and complete ADO of one allele (Figure 3, C and D).

**Figure 4:**
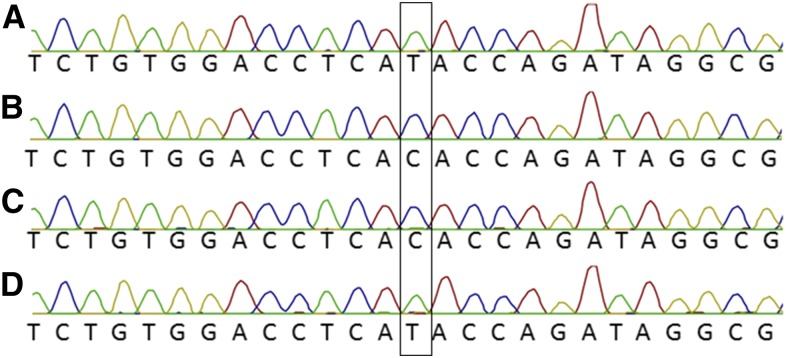
Methylation-dependent restriction digest performed on genomic *PLAGL1* DNA prior to PCR. Genotyping result obtained from PCR amplification of DNA sample NA20588 after treatment with *Hpa*II (A) or McrBC (B). Genotyping result obtained from PCR amplification of DNA sample NA19312 after treatment with *Hpa*II (C) or McrBC (D).

To verify the methylation status of alleles of DNA samples NA19312 and NA20588, we performed methylation-dependent and methylation-sensitive restriction digests on genomic DNA prior to PCR, using the enzymes McrBC and *Hpa*II. McrBC cuts at every methylated CpG dinucleotide, whereas the methylation-sensitive endonuclease *Hpa*II only cuts nonmethylated DNA. After digestion, the DNA was amplified by PCR and Sanger sequenced, enabling the visualization of methylated and nonmethylated DNA in separate experiments. This assay demonstrated that in each case, the methylated allele of genomic DNA dropped out of PCR when using buffer containing 50 mM KCl (Figure 4).

### CD spectroscopy

**Figure 5:**
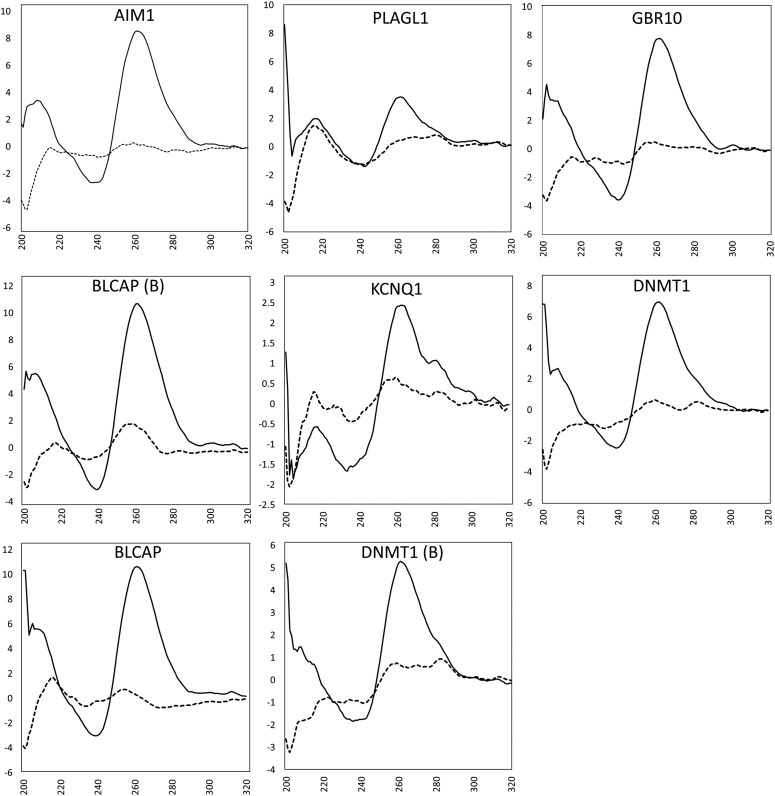
CD spectra of G4 oligonucleotides at 95°. Molar ellipticity (×10^5^ degrees per cm^2^ per dmol^−1^) is on the vertical axis and wavelength (nanometer) is on the horizontal axis. Solid lines represent CD spectra in the presence of 10 mM Tris-HCl, 50 mM KCl, and 1.5 mM MgCl_2_, and dashed lines represent CD spectra in 10 mM Tris-HCl and 1.5 mM MgCl_2_.

To confirm that all putative G4 motifs studied here were capable of forming non-B DNA structures, oligonucleotides corresponding to the predicted G4 sequences were subjected to CD spectroscopy (Figure 5). Structures were assessed at temperatures and conditions relevant to PCR, by collecting CD spectra at 20° (Figure S4, Figure S5, Figure S6, Figure S7, Figure S8, Figure S9, Figure S10, Figure S11, and Figure S12 in File S1) and 95° (Figure 5), in PCR buffer (50 mM KCl, 1.5 mM MgCl_2_, and 10 mM Tris-HCl). G4 formation was inferred by comparison with the equivalent spectrum obtained in the absence of KCl (1.5 mM MgCl_2_ and 10 mM Tris-HCl), which served as the negative control.

In the presence of KCl, all oligonucleotides demonstrated a CD profile which was representative of parallel-stranded G4 formation (Figure 5), consisting of a trough at 245 nm and a peak at 265 nm. However, KCNQ1 and PLAGL1 had additional minor peaks at 295 nm, which suggested the presence of antiparallel G4. All eight oligonucleotides demonstrated stable formation of parallel-stranded G4 at 95°, indicating that structural formation is likely to persist throughout PCR. During PCR, an initial denaturation stage of 2 min at 94° is required to activate the Taq polymerase. Each subsequent cycle involves an additional stage at 94°, which is required to denature double-stranded DNA, prior to primer annealing. The thermal stability of these structures suggests that G4 are likely to be maintained throughout several cycles of PCR. The CD profiles representing the two G4 motifs from *PLAGL1* in genomic DNA are presented in Figure S4 in File S1.

The structural profiles obtained in the presence and absence of potassium demonstrated a cationic dependence for structure formation, a property which is characteristic of G4 (Neidle 2009; Sun and Hurley 2010; Takahama *et al.* 2011; Yang and Hurley 2006). This observation was most pronounced at 95°, where the only structural signatures in the presence of potassium were representative of parallel-stranded G4. Previous analysis indicated ADO is not likely to result from differences in G4 stability between methylated and nonmethylated structures (Stevens *et al.* 2014), and further investigation into the precise mechanism by which G4 and cytosine methylation interact to cause ADO of methylated alleles is required.

### Conclusion

G4 structures are widespread throughout the human genome, and recently, Chambers *et al.* (2015) demonstrated the formation of >716,000 G4 structures in human genomic DNA, using an *in vitro* assay based on polymerase extension and next generation sequencing. We initially described parent-of-origin specific ADO that occurred during amplification of the imprinted human *MEST* promoter region. This ADO was found to result from the combination of cytosine methylation and G4 formation in the template DNA, which presumably blocks amplification by Taq polymerase. In this report we demonstrate, using synthetic templates to mimic genomic DNA, that many other regions of imprinted genes spanning G4-forming motifs are prone to ADO. We then showed that native, differentially methylated genomic DNA from the promoter of the human *PLAGL1* locus displays the same type of parent-of-origin specific ADO of the methylated (paternal) allele first observed at the *MEST* locus (Stevens *et al.* 2014). Our current analysis confirms that parent-of-origin specific ADO is a more general property of many imprinted genes, and highlights the potential for unrecognized genotyping errors in such regions. Therefore, the possibility of systematic genotyping error arising from G4 structures in differentially methylated regions of the genome is an important consideration for the design and application of PCR in diagnostic and research settings. The assay format described here should also prove useful for assessing the propensity of any such genomic region to undergo this type of ADO.

## Supplementary Material

Supplemental material is available online at www.g3journal.org/lookup/suppl/doi:10.1534/g3.116.038687/-/DC1.
