# Supplementary material for: Allelic Dropout During Polymerase Chain Reaction due to G-Quadruplex Structures and DNA Methylation Is Widespread at Imprinted Human Loci
Source: G3 (Bethesda). 2017 Jan 30;7(3):1019–25. doi: 10.1534/g3.116.038687 (PMC5345703; doi:10.1534/g3.116.038687)
Supplement: Supplementary file 1 [file 1019file001.docx]

**Supplementary information**

Table S1. Oligonucleotides used

| Primer Name | Sequence 5’ to 3’* | Experimental use |
| --- | --- | --- |
| pf AIM1  pr AIM1  pfAIM1 SNP  G4AIM1  BSpf AIM1 all  BSpr AIM1 all  BSpf2 AIM1 CG | CACCTCTCAGTGATAAAGGAT  TGCACCACTCAGTCTTTCA  CACCTCTCAGTGATAAAGGATAAGTT  C**GGG**G**GGG**ATGG**GGG**GTGGTA**GGG**  AAAGTTATTTGAAAGTTAATGTTATGG  AAAAGTAACTAATAACAACCAACATCT  ATAAAGGATAGGTTTTTAGGTCG | Template Mixing  Template Mixing and Sequencing  Primer Mutagenesis  CD Spectroscopy  Bisulfite PCR amplification  Bisulfite PCR amplification  Bisulfite methylation specific PCR amplification |
| pfBLCAP  prBLCAP  pfBLCAP SNP  G4BLCAP  BSpf BLCAP all  BSpr BLCAP all  BSpf2 BLCAP CG | TTGCAGGATGAGACAGGCAG  TCAGCCTATCACCCCAGACA  TTGCAGGATGAGACAGGCAGAACC  **GGGG**AGGTG**GGGG**GACGT**GGGG**GTGA**GGGG**  TTGTAGGATGAGATAGGTAGAGT  ATACTTCCTTTCCAAACCATTA  TTGTAGGATGAGATAGGTAGAGTCG | Template Mixing  Template Mixing and Sequencing  Primer Mutagenesis  CD Spectroscopy  Bisulfite PCR amplification  Bisulfite PCR amplification  Bisulfite methylation specific PCR amplification |
| pfBLCAP (B)  pr BLCAP (B)  pfBLCAP (B) SNP  G4BLCAPB  BSpf BLCAP (B) all  BSpr BLCAP (B) all  BSpf2 BLCAP (B) CG | CAACGTGTCTCTGGGGCATA  TCGGGGAGCAGGTTTGCTGT  CAACGTGTCTCTGGGGCATATAAAGGA  **GGGG**GGTG**GGGG**CGGT**GGGG**G**GGGG**  GGGTATATAGAGGAAGTTAGAGGTTAT  CCATTTACTAGATATCTATTTTGAAAT  GTATATAGAGGAAGTTAGAGGTTATCG | Template Mixing  Template Mixing and Sequencing  Primer Mutagenesis  CD Spectroscopy  Bisulfite PCR amplification  Bisulfite PCR amplification  Bisulfite methylation specific PCR amplification |
| pfDNMT1  prDNMT1  pfDNMT1 SNP**  G4DNMT1  BSpfDNMT1all  BSprDNMT1all  BSpf2DNMT1CG | GGCAGAAGTCCTTCCTTCCC  AGCCTCATTCCCATCAAGTAGC  GGCAGAAGTCCTTCCTTCCCAAAT  C**GGG**GGCTG**GGG**GCTGA**GGG**CCGGTT**GGG**  GTAGGTGTTTGAGATTTATGTT  AACATTTCTATCACTCAAATATTA  TATTTGGATAGTGGGGTCG | Template Mixing  Template Mixing and Sequencing  Primer Mutagenesis  CD Spectroscopy  Bisulfite PCR amplification  Bisulfite PCR amplification  Bisulfite methylation specific PCR amplification |
| pfDNMT1 (B)  prDNMT1 (B)  pfDNMT1 (B) SNP  G4DNMT1B  BSpfDNMT1 (B) all  BSprDNMT1 (B) all  BSpf2DNMT1 (B) CG | ACAGTGGGTCGTTTCTTCCC  TTCAGTAAGGGATGGCTGGC  ACAGTGGGTCGTTTCTTCCCTATGTCT  **GGG**CCCT**GGG**GCT**GGG**GC**GGG**  TGATAATATTTGTGAGGGGTTTT  CTATACCATTAAACCCTACCTTCA  TGATAATATTTGTGAGGGGTTTTCG | Template Mixing  Template Mixing and Sequencing  Primer Mutagenesis  CD Spectroscopy  Bisulfite PCR amplification  Bisulfite PCR amplification  Bisulfite methylation specific PCR amplification |
| pfGRB10  prGRB10  pfGRB10 SNP  G4GRB10  BSpf GRB10all  BSpr GRB10all  BSpf2 GRB10CG | GTTTGGGAAACTGCCTGCAG  GCAACAGATGAGAACAGTGG  GTTTGGGAAACTGCCTGCAGAAAG  **GGG**T**GGGG**GA**GGG**CT**GGG**  TAGTTTATATGTTTATTTGTAGATTTAG  ATCAACTTTAAACATTACAAATATAC  TAGTTTATATGTTTATTTGTAGATTTAGCG | Template Mixing  Template Mixing and Sequencing  Primer Mutagenesis  CD Spectroscopy  Bisulfite PCR amplification  Bisulfite PCR amplification  Bisulfite methylation specific PCR amplification |
| pfKCNQ1  prKCNQ1  pfKCNQ1 SNP  G4KCNQ1  BSpf KCNQ1all  BSpr KCNQ1all  BSpf2 KCNQ1CG | CCTGGCTTCGGTCCCCTGGA  GCAGTGCCCAGCACAGGGAG  CCTGGCTTCGGTCCCCTGGACTGG  C**GGGG**ACT**GGGG**CT**GGGG**CT**GGGG**  TGTTTTTGGTGTAGGTGG  CCTAAACACCCACAAACCT  TTTTTGGTGTAGGTGGCG | Template Mixing  Template Mixing and Sequencing  Primer Mutagenesis  CD Spectroscopy  Bisulfite PCR amplification  Bisulfite PCR amplification  Bisulfite methylation specific PCR amplification |
| pfPLAGL1  prPLAGL1  pfPLAGL1 SNP  G4PLAGL1  PLAGL1Fa  PLAGL1Ra  G4PLAGL1a  G4PLAGL1b  BSpf PLAGL1all  BSpr PLAGL1all  BSpf2 PLAGL1 | AGAGCAAACTCGGCAGGCGG  AGAGCAAACTCGGCAGGCGG  AGAGCAAACTCGGCAGGCGGATTA  **GGG**GTTG**GGG**GAAA**GGG**GTTT**GGG**  CCATAAGAGACGAAAGTGCAAACA  CGAGGAGGGTGTGCCTTTG  GGGGCTGCCTGGGTTGCGGGTGACGATCTGCGGGG  CGGGTCCGGGCTCCGCGGGGCCGGGTGCGGG  ATAGATTATGATATTTAGTAGAGTAAACT  CAAATACCCCTCACCATCCT  ATAGATTATGATATTTAGTAGAGTAAACTCG | Template Mixing  Template Mixing and Sequencing  Primer Mutagenesis  CD Spectroscopy  PCR amplification genomic DNA  PCR amplification genomic DNA  CD Spectroscopy of genomic G4a  CD Spectroscopy of genomic G4b  Bisulfite PCR amplification  Bisulfite PCR amplification  Bisulfite methylation specific PCR amplification |

*Bold nucleotides indicate guanines predicted by QGRS Mapper to contribute towards G4 formation.

**Underlined nucleotides denote the position of the artificial SNP introduced by primer mutagenesis


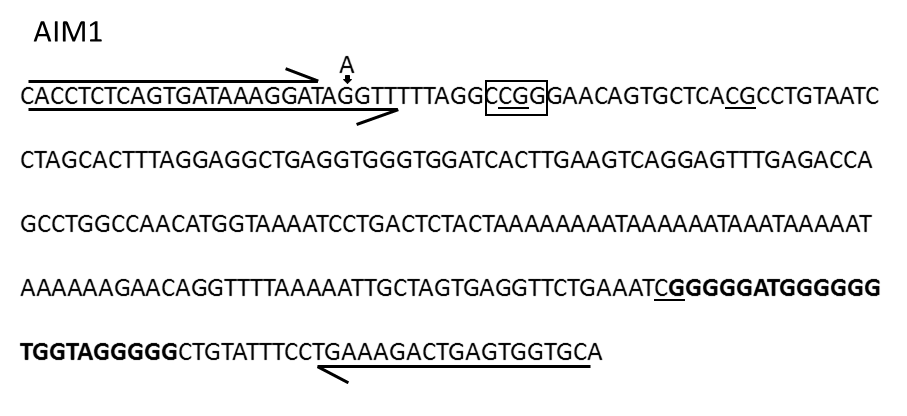

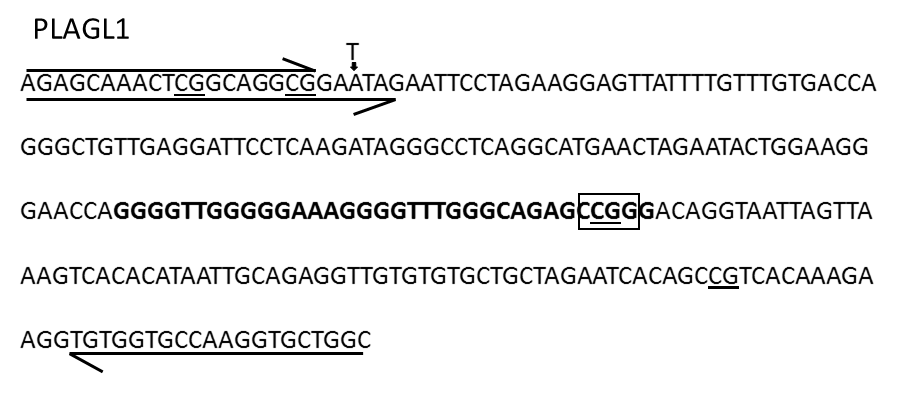

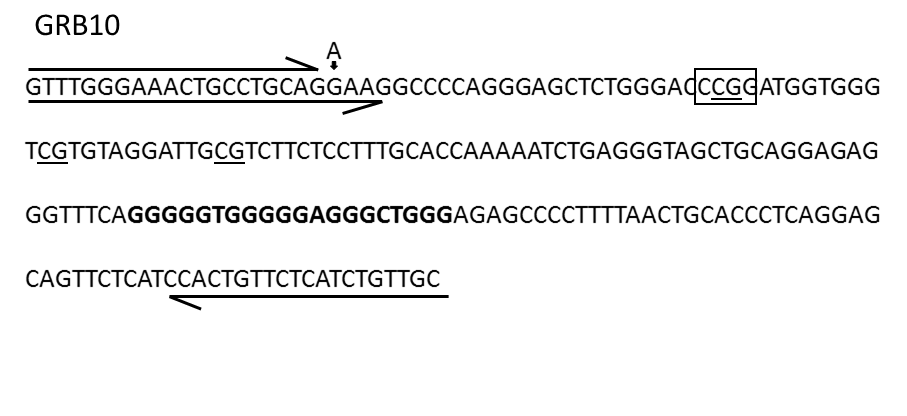

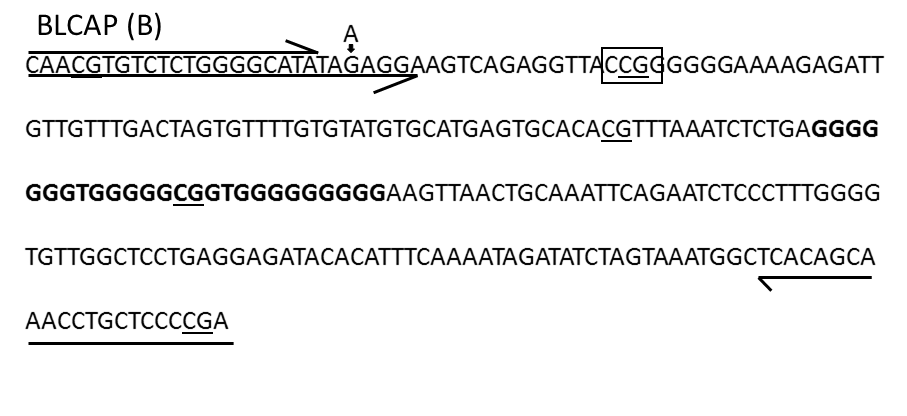

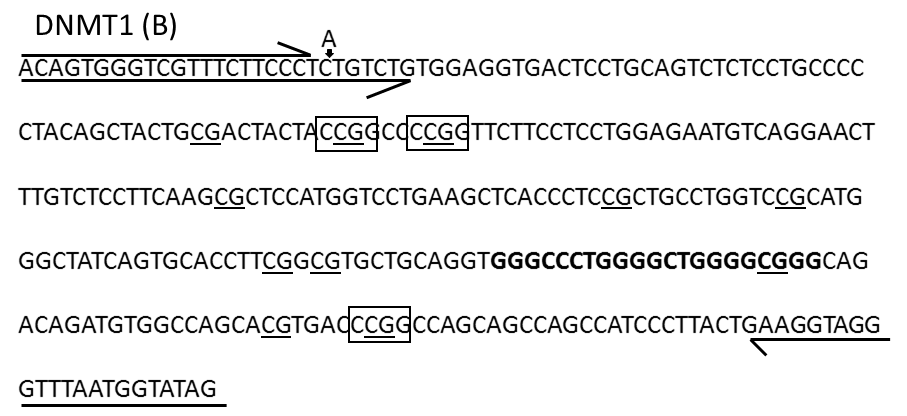

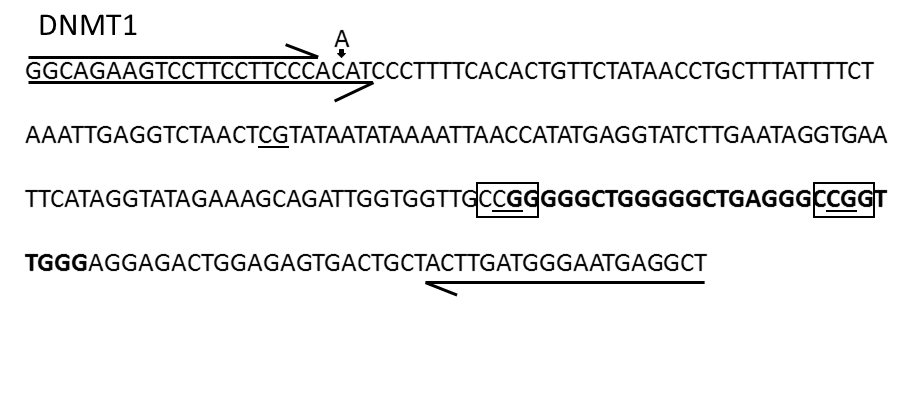

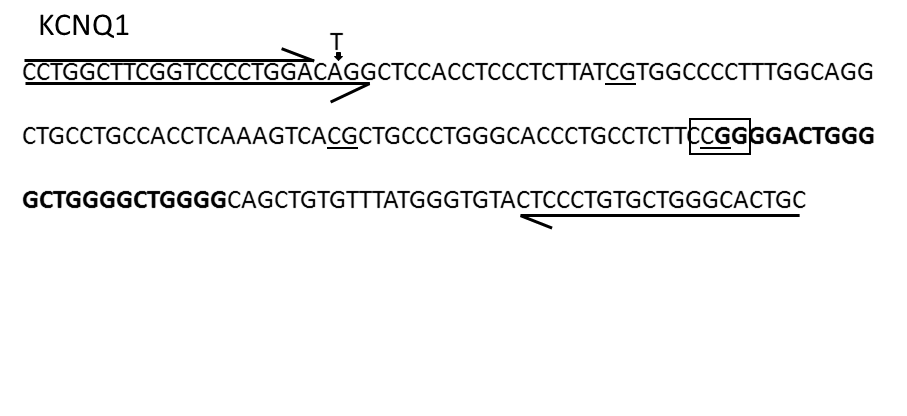

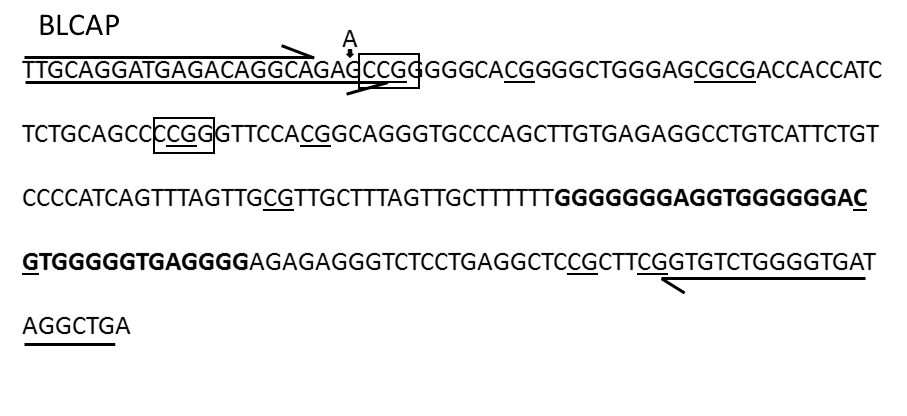


**Figure S1. DNA sequences of PCR amplicons used in template mixing experiments.**

Horizontal black arrows represent the forward and reverse primers used for amplification. The longer forward arrow represents the primer used during primer mutagenesis. The single nucleotide above the vertical black arrows represents the nucleotide substituted during primer mutagenesis. Underlined bases represent CpG dinucleotides which can be potentially methylated using M. SssI. Bold bases represent the G4 forming motifs. Black box indicates the recognition sequence for HpaII/MspI , used for assessing successful methylation.

23


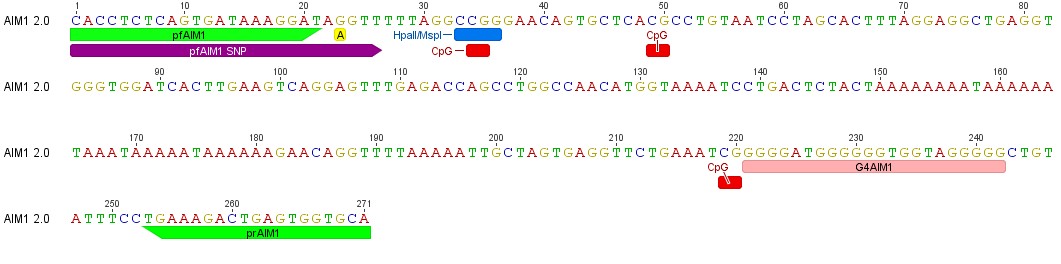


**Figure S2. Example of primer combinations used for primer mutagenesis and amplification.**

The pfAIM1 SNP primer (purple) was used to generate a synthetic template containing an “A” allele (yellow) at the 23^rd^ nt in place of a “G”. The alternative template was generated using pfAIM1 primer (green), and consisted of the wild-type “G” containing sequence. Re-amplification during the subsequent mixing experiments was performed using the pfAIM1 primer, and a common reverse primer (Supplementary Table 1.). Genotyping of products was performed by Sanger sequencing with the reverse primer.


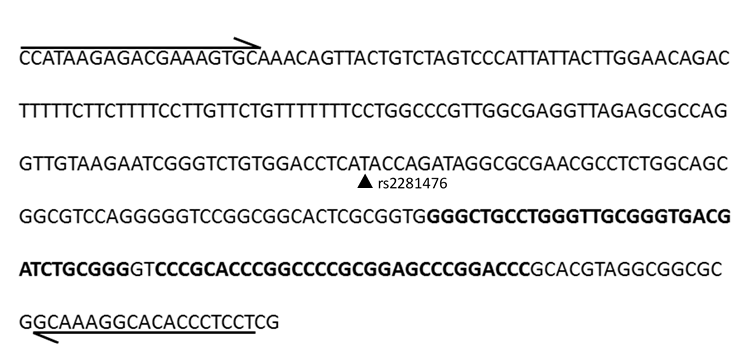


**Figure S3. Genomic DNA sequences of *PLAGL1* used to demonstrate ADO in genomic DNA**

Horizontal black arrows represent the forward and reverse primers used for amplification. Bold bases represent the G4 forming motifs.


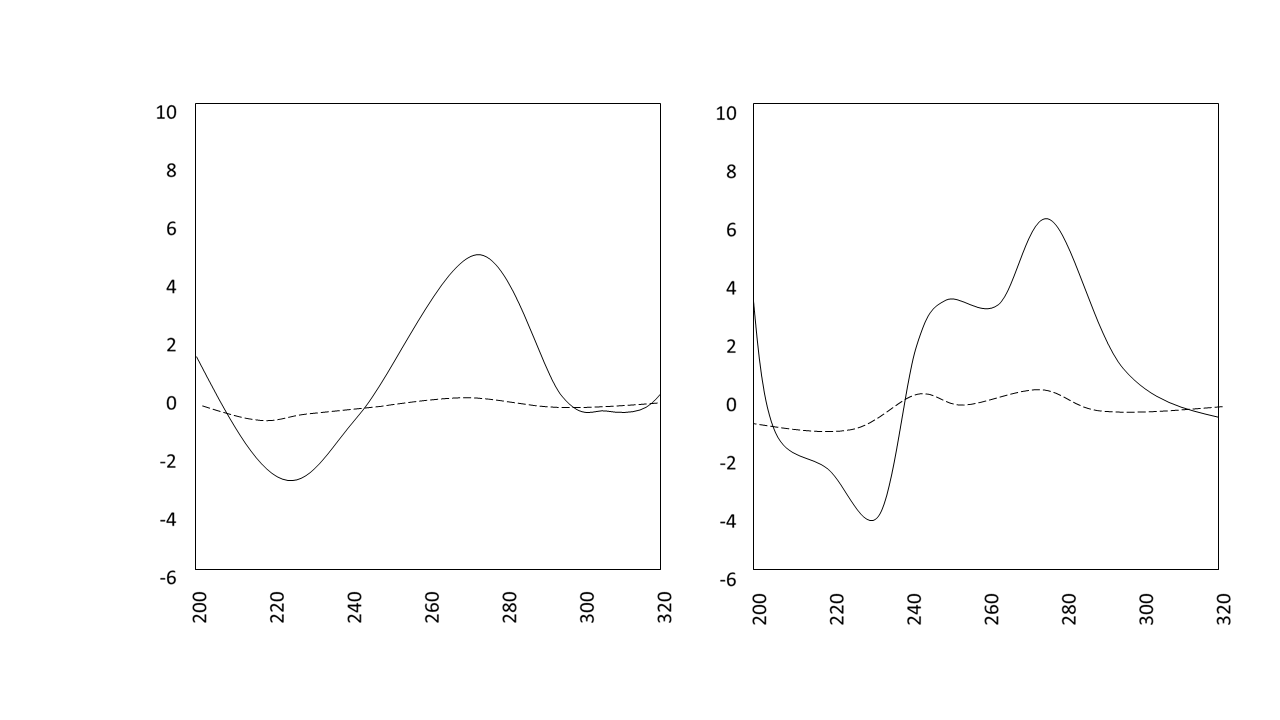


A B

**Figure S4. CD spectra of PLAGL1 G4 from genomic DNA at 20^o^C and 95^o^C.**

(A) G4PLAGL1a (B) G4PLAGL1b. Molar ellipticity (x10^5^ deg.cm^2^.dmol^-1^) is on the vertical axis and wavelength (nm) is on the horizontal axis. Solid lines represent CD spectra in the presence of 10 mM Tris-HCl 50 mM KCl and 1.5 mM MgCl_2_, and the dashed lines represent CD spectra in 10 mM Tris-HCl and 1.5 mM MgCl_2_. DNA sequences of G4PLAGL1a and G4PLAGL1b are shown in Supplementary Figure 2 and Supplementary Table 1.

**Figure S5. CD spectra of AIM1 G4 at 20^o^C and 95^o^C.**

Spectral profiles obtained at 20^o^C (grey lines) and 95^o^C (black lines), in PCR buffer. Samples analysed in 10 mM Tris-HCl, 50 mM KCl and 1.5 mM MgCl_2_ are represented by solid lines and samples analysed in 10 mM Tris-HCl and 1.5 mM MgCl_2_ are represented by dashed lines. Molar ellipticity (x10^5^ deg.cm^2^.dmol^-1^) is on the vertical axis and wavelength (nm) is on the horizontal axis.

**Figure S6. CD spectra of BLCAP G4 at 20^o^C and 95^o^C.**

Spectral profiles obtained at 20^o^C (grey lines) and 95^o^C (black lines), in PCR buffer. Samples analysed in 10 mM Tris-HCl, 50 mM KCl and 1.5 mM MgCl_2_ are represented by solid lines and samples analysed in 10 mM Tris-HCl and 1.5 mM MgCl_2_ are represented by dashed lines. Molar ellipticity (x10^5^ deg.cm^2^.dmol^-1^) is on the vertical axis and wavelength (nm) is on the horizontal axis.

**Figure S7. CD spectra of BLCAP (B) G4 at 20^o^C and 95^o^C**

Spectral profiles obtained at 20^o^C (grey lines) and 95^o^C (black lines), in PCR buffer. Samples analysed in 10 mM Tris-HCl, 50 mM KCl and 1.5 mM MgCl_2_ are represented by solid lines and samples analysed in 10 mM Tris-HCl and 1.5 mM MgCl_2_ are represented by dashed lines. Molar ellipticity (x10^5^ deg.cm^2^.dmol^-1^) is on the vertical axis and wavelength (nm) is on the horizontal axis.

**Figure S8. CD spectra of DNMT1 G4 at 20^o^C and 95^o^C.**

Spectral profiles obtained at 20^o^C (grey lines) and 95^o^C (black lines), in PCR buffer. Samples analysed in 10 mM Tris-HCl, 50 mM KCl and 1.5 mM MgCl_2_ are represented by solid lines and samples analysed in 10 mM Tris-HCl and 1.5 mM MgCl_2_ are represented by dashed lines. Molar ellipticity (x10^5^ deg.cm^2^.dmol^-1^) is on the vertical axis and wavelength (nm) is on the horizontal axis.

**Figure S9. CD spectra of DNMT1 (B) G4 at 20^o^C and 95^o^C.**

Spectral profiles obtained at 20^o^C (grey lines) and 95^o^C (black lines), in PCR buffer. Samples analysed in 10 mM Tris-HCl, 50 mM KCl and 1.5 mM MgCl_2_ are represented by solid lines and samples analysed in 10 mM Tris-HCl and 1.5 mM MgCl_2_ are represented by dashed lines. Molar ellipticity (x10^5^ deg.cm^2^.dmol^-1^) is on the vertical axis and wavelength (nm) is on the horizontal axis.

**Figure S10. CD spectra of GRB10 G4 at 20^o^C and 95^o^C.**

Spectral profiles obtained at 20^o^C (grey lines) and 95^o^C (black lines), in PCR buffer. Samples analysed in 10 mM Tris-HCl, 50 mM KCl and 1.5 mM MgCl_2_ are represented by solid lines and samples analysed in 10 mM Tris-HCl and 1.5 mM MgCl_2_ are represented by dashed lines. Molar ellipticity (x10^5^ deg.cm^2^.dmol^-1^) is on the vertical axis and wavelength (nm) is on the horizontal axis.

**Figure S11. CD spectra of KCNQ1 G4 at 20^o^C and 95^o^C.**

Spectral profiles obtained at 20^o^C (grey lines) and 95^o^C (black lines), in PCR buffer. Samples analysed in 10 mM Tris-HCl, 50 mM KCl and 1.5 mM MgCl_2_ are represented by solid lines and samples analysed in 10 mM Tris-HCl and 1.5 mM MgCl_2_ are represented by dashed lines. Molar ellipticity (x10^5^ deg.cm^2^.dmol^-1^) is on the vertical axis and wavelength (nm) is on the horizontal axis.

**Figure S12. CD spectra of PLAGL1 G4 at 20^o^C and 95^o^C.**

Spectral profiles obtained at 20^o^C (grey lines) and 95^o^C (black lines), in PCR buffer. Samples analysed in 10 mM Tris-HCl, 50 mM KCl and 1.5 mM MgCl_2_ are represented by solid lines and samples analysed in 10 mM Tris-HCl and 1.5 mM MgCl_2_ are represented by dashed lines. Molar ellipticity (x10^5^ deg.cm^2^.dmol^-1^) is on the vertical axis and wavelength (nm) is on the horizontal axis.
