# Supplementary material for: Allelic Dropout During Polymerase Chain Reaction due to G-Quadruplex Structures and DNA Methylation Is Widespread at Imprinted Human Loci
Source: G3 (Bethesda). 2017 Jan 30;7(3):1019–25. doi: 10.1534/g3.116.038687 (PMC5345703; doi:10.1534/g3.116.038687)
Supplement: Supplementary file 2 [file 1019file002.docx]

**Allelic drop-out during polymerase chain reaction due to G-quadruplex structures and DNA methylation is widespread at imprinted human loci**

Aaron J. Stevens^1^, Millie G. Taylor^1^, F. Grant Pearce^2^, Martin A. Kennedy^1*^

^1^ Department of Pathology, University of Otago Christchurch, P.O. Box 4345, Christchurch, New Zealand.

^2^ Biomolecular Interaction Centre, School of Biological Sciences, University of Canterbury, Christchurch, New Zealand.

**Running title:** Allelic drop-out due to G4 and methylation

**Correspondence to:**

Prof M A Kennedy, Department of Pathology, University of Otago, Christchurch, P.O. Box 4345, Christchurch, New Zealand. Phone +6433640587. Email martin.kennedy@otago.ac.nz

**ABSTRACT**

Loss of one allele during polymerase chain reaction (PCR) amplification of DNA, known as allelic drop-out, can be caused by a variety of mechanisms. Allelic drop-out during PCR may have profound implications for molecular diagnostic and research procedures that depend on PCR and assume biallelic amplification has occurred. Complete allelic drop-out due to the combined effects of cytosine methylation and G-quadruplex formation was previously described for a differentially methylated region of the human imprinted gene, *MEST*. We now demonstrate that this parent-of-origin specific allelic drop-out can potentially occur at several other genomic regions that display genomic imprinting and have propensity for G-quadruplex formation, including *AIM1*, *BLCAP*, *DNMT1*, *PLAGL1*, *KCNQ1* and *GRB10*. These findings demonstrate that systematic allelic drop-out during PCR is a general phenomenon for regions of the genome where differential allelic methylation and G-quadruplex motifs coincide, and suggest that great care must be taken to ensure biallelic amplification is occurring in such situations.

**INTRODUCTION**

DNA amplification by polymerase chain reaction (PCR) is an enzymatic technique for the *in vitro* synthesis of targeted DNA regions, mediated by thermally stable DNA polymerases. PCR is a prerequisite for most experimental procedures that involve DNA detection, sequencing, cloning and genotyping (Mullis and Faloona 1987; Saiki *et al.* 1988; Bevan *et al.* 1992) and is of widespread application in genetic research and molecular diagnostic applications (Desforges and Eisenstein 1990). Despite extensive optimisation and near ubiquitous usage, PCR is still prone to failure under certain circumstances. For diploid organisms, the failure of one allele to amplify can result in allelic drop-out (ADO), causing apparent homozygosity (Askree *et al.* 2011; Boán *et al.* 2004; Lam and Mak 2013; Landsverk *et al.* 2012; Piyamongkol *et al.* 2003; Saunders *et al.* 2010a; Wenzel *et al.* 2009). ADO is an insidious problem that is difficult to recognise because the PCR appears successful, but half of the genetic information is missing. ADO can have significant implications in both research and clinical applications, where there is a requirement for high sensitivity and accurate PCR genotyping. Incorrect genotyping can have substantial negative consequences and may result in the misdiagnosis of genetic diseases, loss of the ability to differentiate between individuals, and false assumptions about parentage or genetic diversity (Boán *et al.* 2004; Landsverk *et al.* 2012; Saunders *et al.* 2010b; Tomaz *et al.* 2010; Wenzel *et al.* 2009).

We previously characterised a novel mechanism of ADO which occurred during genotyping of the imprinted human gene, *MEST* (Stevens *et al.* 2014). Extensive PCR analysis of a short region in the *MEST* promoter invariably led to non-Mendelian genotype patterns for three single nucleotide polymorphisms (SNPs), which could not be resolved by primer redesign, or standard PCR optimisation strategies. We established that both cytosine methylation and DNA structures known as G-quadruplexes (G4) in this region contributed to ADO, leading to incorrect genotyping (Stevens *et al.* 2014). G4 are secondary DNA structures that can form in G-rich regions due to the self-association of guanine through Hoogsteen bonds. Four guanine nucleotides can adopt a square planar arrangement referred to as a G-quartet, and multiple G-quartets can then stack upon one another to form a G4 (Sen and Gilbert 1988; Sundquist and Klug 1989). G4 formation is stabilised by the integration of a cation, like potassium (Ambrus *et al.* 2005; Biffi *et al.* 2012; Maizels 2015; Rhodes and Lipps 2015; Simonsson 2001), making PCR buffer an optimal environment for G4 formation. G4 structures may then act as a steric block to Taq polymerase (Boán *et al.* 2004; Chambers *et al.* 2015; Saunders *et al.* 2010b; Weitzmann *et al.* 1996), an effect that is exacerbated when the G4 region is methylated (Stevens *et al.* 2014).

During amplification of the imprinted *MEST* promoter region, we observed consistent ADO of the maternally inherited, methylated allele. Correct genotypes from this locus were only obtained using extraordinary modifications of PCR, including methylation-specific PCR, allele-specific enzymatic digestion of genomic DNA, or PCR buffers lacking potassium. We demonstrated that ADO resulted from the combination of both cytosine methylation and guanine Hoogsteen bonds in the template DNA, which can form G4 structures, and that neither factor in isolation was sufficient to cause complete ADO (Stevens *et al.* 2014).

The novel form of ADO observed at the *MEST* promoter region was intriguing and problematic, but it was unclear if it was a phenomenon restricted solely to this genomic region, or a more general occurrence throughout the genome. In this report we describe the design of an assay and its application to test for the potential occurrence of ADO during amplification of differentially methylated DNA. The templates used in this assay were generated by PCR from a range of imprinted genes, and *in vitro* methylation with the enzyme M. SssI was used to mimick differentially methylated alleles. After demonstrating potential ADO using the assay on multiple synthetic model templates, we then demonstrated that this type of ADO could be observed in genomic DNA analysis of an imprinted human locus other than *MEST*.

**MATERIALS AND METHODS**

**Selection of G-quadruplexes for analysis**

Imprinted genes with a confirmed parent-of-origin methylation status (Jirtle 2012; Morison *et al.* 2001) were analysed for G4 forming motifs using the bioinformatic software, QGRS Mapper (Oleg Kikin 2006). G4 motifs that contained runs of three or more guanines, and a loop length between 0 and 7 nt were considered for analysis, as these were most likely to adopt G4 structure. This corresponded with a QGRS mapper score of at least 37, which was therefore selected as an arbitrary threshold for G4 cut off. PCR amplicons of approximately 300bp were designed from these regions, to contain a single G4 motif and a CCGG endonuclease recognition sequence for HpaII and MspI endonucleases (Table S1 and Fig. S1).

**Polymerase chain reaction (PCR)**

PCR was carried out in a Mastercycler pro thermal cycler (Eppendorf, Stevenage, UK) with Fisher Taq-ti polymerase (Fisher Biotec, Wembley WA, Australia). The initial denaturation step consisted of 95°C for 2 min, and extension was performed at 72°C. Cycling conditions consisted of denaturation at 95°C for 15 secs, annealing for 15 secs, and extension for 45 secs. The initial annealing temperature was 65°C, and this was decreased by 1°C per cycle for 10 cycles, followed by 25 cycles at 55°C. A final extension was performed for 5 min.

**Sanger DNA sequencing**

PCR products were prepared for Sanger DNA sequencing by purification using AcroPrep (PALL Corporation, New York, USA) 96 well filter plates (omega 30K). Purified PCR amplicons were then resuspended in water and ∼10 ng was sequenced with the appropriate primer, using BigDye Terminator v3.1 Cycle Sequencing Kit (Applied Biosystems, Foster City, CA, USA), following the manufacturer's protocol. Sequencing reaction products were run on an AB3130xl fragment analysis system equipped with a 50 cm capillary using POP7 polymer.

**Synthetic DNA templates**

Selected G4-containing amplicons (Fig. S1) were amplified by PCR, and as described below (Allelic drop-out assay) a single nucleotide difference was introduced to allow “alleles” to be distinguished. *In vitro* methylation and digestion experiments were performed on PCR products generated from genomic DNA, using enzymes purchased from New England Biolabs Inc, (Ipswich, MA, USA). *In vitro* methylation was carried out by incubation with M.SssI for 120 mins at 37°C followed by heat inactivation at 65°C for 20 mins, as recommended by the manufacturer's protocol. To assess the extent of *in vitro* methylation achieved, each methylated DNA template was then incubated with the restriction enzyme MspI and its methylation-sensitive isoschizomer HpaII. The resulting digestion products were analysed by gel electrophoresis and compared, to ensure successful methylation (data not shown).

**Allelic drop-out assay on synthetic templates**

The assay for modelling methylation specific allelic drop-out was based on the method described by Stevens *et al.* (2014) (Stevens *et al.* 2014). Synthetic DNA templates generated by PCR of genomic DNA were used for this assay because the endogenous regions did not contain the necessary SNPs for detecting ADO. During PCR an artificial SNP was introduced by primer mutagenesis (Simsek and Adnan 2000) to create two “alleles”, which could be distinguished by Sanger sequencing (Fig. 1A) (Fig. S1 and S2). This meant that for each amplicon there was a “wild type” and “mutant” template that differed by a single base pair near the 3’ end of the forward primer (Fig. S2). Aliquots of each synthetic template were then subjected to *in vitro* methylation with M.SssI (as described above), to produce four different synthetic template types, which were A methylated, A non-methylated, G methylated, and G non-methylated (Fig. 1B).

Methylated and non-methylated amplicons were mixed in various combinations and used to seed PCR, the products of which were then genotyped by Sanger sequencing. For each region, three different mixing experiments were performed, two consisting of reciprocal pairs of methylated and non-methylated templates (mimicking maternal or paternal alleles for imprinted genomic DNA), and one consisting of both non-methylated templates (Fig. 1B). Mixing of the non-methylated templates was a control to ensure that introducing an artificial SNP into the DNA sequence did not influence the genotyping outcome. Each assay was repeated at least two times, for each gene region.

**Allelic drop-out during genomic DNA analysis of *PLAGL1***

Genomic *PLAGL1* analysis was performed on DNA samples NA19312 and NA20588 (Coriell Institute for Medical Research, New Jersey, USA) that were known to be heterozygous at SNP rs2281476. PCR amplification using low potassium PCR buffer (Stevens *et al.* 2014), followed by Sanger sequencing, was used to detect heterozygosity at SNP rs2281476. To demonstrate ADO, genotypes obtained in low potassium buffer were compared to genotypes obtained in standard PCR buffer (containing 50 mM KCl).

The methylation status of each allele in genomic DNA for the *PLAGL1* amplicon was interrogated using methylation-dependent digestion by McrBC and HpaII endonucleases (New England Biolabs Inc., Ipswich, MA, USA). This was performed on ∼70 ng genomic DNA according to the manufacturer's protocol. PCR was then separately performed on the differentially digested genomic DNA aliquots and the amplicons were analysed by Sanger sequencing. To determine the methylation status of the allele that failed to visualise, the genotyping results obtained in this way were compared to those derived from standard PCR performed on non-digested genomic DNA of known haplotypes.

**Circular dichroism spectroscopy**

Oligonucleotides were purchased from Integrated DNA Technologies (IDT Pte. Ltd., Singapore) and assessed for G4 formation in 10 mM Tris-HCl, 1.5 mM MgCl_2_ (pH 7), in the presence and absence of 50 mM KCl. 4 µM of oligonucleotide was heated at 95^o^C for 10 minutes then cooled slowly to room temperature (22^o^C) overnight. Circular dichroism (CD) measurements were performed on a J-815 CD Spectrometer (Jasco Analytical Instruments, MD, USA), using a 1 mm path length quartz cuvette. Spectra were collected across 340 nm to 200 nm in 1 nm increments at both 25^o^C and 95^o^C. The reported spectra corresponded to the average of three scans, and an appropriate buffer blank was made for all spectra.

**Data Availability**

File S1 contains detailed descriptions of all DNA sequences, oligonucleotide sequences and original CD spectroscopy data.

**RESULTS AND DISCUSSION**

**Modelling allelic drop-out with synthetic templates**

We previously demonstrated that the co-localisation of methylation with G4 structure results in ADO during PCR amplification of the human *MEST* promoter region. This was caused by both G4 formation and cytosine methylation in the DNA template, with neither factor alone being sufficient to cause ADO (Stevens *et al.* 2014). To determine the potential for more widespread occurrence of this phenomenon, we have now tested G4-motif containing regions of several other imprinted genes. For this purpose we used synthetic templates generated by PCR (with introduction of an artificial SNP to allow differentiation of alleles) followed by *in vitro* methylation. Mixing of these alleles in specific combinations allowed us to mimic monoallelic methylation of genomic DNA derived from several human imprinted genes. Eight regions from six genes (*AIM1, BLCAP, DNMT1, PLAGL1, KCNQ1* and *GRB10*) were investigated using this ADO assay. For each of the genes *DNMT1* and *PLAGL1*, two separate amplicons were studied from the same gene, and the second amplicon is described as DNMT1 (B) or PLAGL1 (B) (Table S1 and Fig. S1).

Each of the eight assayed amplicons demonstrated ADO of the methylated template in at least one of the two reciprocal mixing experiments (Fig. 2). Clear ADO of the methylated allele was always observed in mixing experiments with templates AIM1, PLAGL1, GRB10, BLCAP (B) and DNTM1 (B), with only the non-methylated template detected during genotyping (by Sanger sequencing) (Fig. 2). For these regions, amplification products from the mix of two non-methylated templates gave heterozygous genotypes, with both “alleles” successfully detected in all cases (Fig. 2).

Amplicons from KCNQ1 and DNMT1 demonstrated clear ADO in only one of the two methylated template mixing experiments, with a minor peak from the methylated template visible in the reciprocal mixing experiment for each amplicon, indicating partial ADO (Fig. 2). For these two amplicons, mixing of the non-methylated templates demonstrated a clear heterozygous pattern in the Sanger sequencing traces (Fig. 2). Because methylated and non-methylated templates had identical sequences (except for the introduced artificial SNP), partial ADO during the reciprocal mixing experiment was likely to reflect inefficient methylation by M. SssI. Although the efficiency of methylation was assessed using restriction digest by HpaII and MspI, this can only detect methylated cytosine within the recognition sequence CCGG, and not at additional CpG dinucleotides present in each amplicon (Fig. S1).

Amplicons from BLCAP demonstrated ADO of each methylated template during the mixing experiments, however, when the non-methylated templates were mixed, ADO was also apparent (Fig. 2). This may indicate that the introduction of the artificial SNP decreased the amplification efficiency of the “G” template. However, there was still clear evidence of ADO when each template was methylated.

Many factors may potentially interact to direct ADO during PCR of differentially methylated regions that contain G4 and further research is required to completely understand this process. Amplicon size did not appear to correlate with the propensity for ADO, which predominantly appears to be determined by the position and stability of the G4. We did not determine if the number or position of methylated CpGs also contributed towards ADO.

The data presented here demonstrate that, for regions spanning a G4 motif, amplification of non-methylated DNA was always favoured during PCR, leading to ADO of the methylated allele. This confirmed that parent-of-origin specific ADO in regions of differential methylation that contain a G4 motif is not unique to *MEST*, and is a more general phenomenon likely to occur at many imprinted regions of the genome.

**ADO at the genomic *PLAGL1* locus**

Because the synthetic template assay is a model of differentially methylated DNA, we sought to extend our analysis to an endogenous region of imprinted genomic DNA other than the *MEST* promoter (Stevens *et al.* 2014), where this form of ADO was originally described. The genomic regions that were used to generate synthetic templates for the ADO assay did not contain common, endogenous SNPs, which are necessary for the detection of ADO by Sanger sequencing. However, a region located approximately 200bp upstream of the PLAGL1 (B) amplicon contains a SNP (rs2281476) with a minor allele frequency of ~25% in Europeans (Lek *et al.* 2016). This region is a differentially methylated CpG island associated with the promoter of *PLAGL1* (Yuen et al. 2011; Choufani et al. 2011), and rs2281476 is located 130 bp 5’ of two G4 motifs that are located on opposite DNA strands (Fig. S3). This combination of G4 motif, imprinted methylation and presence of a SNP marked this region as a good target for detection of ADO.

Genomic DNA samples were screened by PCR amplification using low potassium PCR buffer (to prevent G4 formation and ADO) (Stevens *et al.* 2014), followed by Sanger sequencing, to identify individuals heterozygous at rs2281476. Although several heterozygous individuals were identified, results were consistent and data from only two (referred to as DNA sample NA19312 and NA20588) are presented (Fig. 3A and B). Amplification of these genomic DNA samples using standard PCR buffer (50 mM KCl) revealed consistent and complete ADO of one allele (Fig. 3C and D).

To verify the methylation status of alleles of DNA sample NA19312 and NA20588, we performed methylation-dependent and methylation-sensitive restriction digests on genomic DNA, prior to PCR, using the enzymes McrBC and HpaII. McrBC cuts at every methylated CpG dinucleotide, whereas the methylation-sensitive endonuclease HpaII only cuts non-methylated DNA. After digestion the DNA was amplified by PCR and Sanger sequenced, enabling the visualisation of methylated and non-methylated DNA in separate experiments. This assay demonstrated that in each case the methylated allele of genomic DNA dropped out of PCR when using buffer containing 50mM KCl (Fig. 4).

**Circular Dichroism Spectroscopy**

To confirm that all putative G4 motifs studied here were capable of forming non-B DNA structures, oligonucleotides corresponding to the predicted G4 sequences were subjected to CD spectroscopy (Fig. 5). Structures were assessed at temperatures and conditions relevant to PCR, by collecting CD spectra at 20^o^C (Fig. S4-12) and 95^o^C (Fig. 5), in PCR buffer (50 mM KCl, 1.5 mM MgCl_2_ and 10 mM Tris-HCl). G4 formation was inferred by comparison with the equivalent spectrum obtained in the absence of KCl (1.5 mM MgCl_2_ and 10 mM Tris-HCl), which served as the negative control.

In the presence of KCl, all oligonucleotides demonstrated a CD profile which was representative of parallel stranded G4 formation (Fig. 5), consisting of a trough at 245 nm and a peak at 265 nm. However, KCNQ1 and PLAGL1 had additional minor peaks at 295 nm, which suggested the presence of antiparallel G4. All eight oligonucleotides demonstrated stable formation of parallel stranded G4 at 95^o^C, indicating that structural formation is likely to persist throughout PCR. During PCR, an initial denaturation stage of two minutes at 94^o^C is required to activate the Taq polymerase. Each subsequent cycle involves an additional stage at 94^o^C, which is required to denature double stranded DNA, prior to primer annealing. The thermal stability of these structures suggests that G4 are likely to be maintained throughout several cycles of PCR. The CD profiles representing the two G4 motifs from *PLAGL1* in genomic DNA are presented in Fig. S4.

The structural profiles obtained in the presence and absence of K^+^ demonstrated a cationic dependence for structure formation, a property which is characteristic of G4 (Neidle 2009; Sun and Hurley 2010; Takahama *et al.* 2011; Yang and Hurley 2006). This observation was most pronounced at 95^o^C, where the only structural signatures in the presence of K^+^ were representative of parallel stranded G4. Previous analysis indicated ADO is not likely to result from differences in G4 stability between methylated and non-methylated structures (Stevens *et al.* 2014), and further investigation into the precise mechanism by which G4 and cytosine methylation interact to cause ADO of methylated alleles is required.

**CONCLUSION**

G-quadruplex structures are widespread throughout the human genome, and recently Chambers *et al.* (2015) demonstrated the formation of over 716,000 G4 structures in human genomic DNA, using an *in vitro* assay based upon polymerase extension and next generation sequencing (Chambers *et al.* 2015). We initially described parent-of-origin specific ADO which occurred during amplification of the imprinted human *MEST* promoter region. This ADO was found to result from the combination of cytosine methylation and G4 formation in the template DNA, which presumably blocks amplification by Taq polymerase. In this report we demonstrated that many other regions of imprinted genes spanning G4 forming motifs are prone to ADO, by using synthetic templates to mimic genomic DNA. We then showed that native, differentially methylated genomic DNA from the promoter of the human *PLAGL1* locus displays the same type of parent-of-origin specific ADO of the methylated (paternal) allele first observed at the *MEST* locus (Stevens *et al.* 2014). Our current analysis confirms that parent-of-origin specific ADO is a more general property of many imprinted genes, and highlights the potential for unrecognised genotyping errors in such regions. Therefore, the possibility of systematic genotyping error arising from G-quadruplex structures in differentially methylated regions of the genome is an important consideration for the design and application of PCR in diagnostic and research settings. The assay format described here should also prove useful for assessing the propensity of any such genomic region to undergo this type of ADO.

**ACKNOWLEDGEMENTS**

We are grateful to Hannah Kennedy (Canterbury Health Laboratories) for support with DNA sequencing analysis. We are also grateful to the anonymous reviewers who provided astute and constructive feedback. This research was supported by the Marsden Fund Council from New Zealand Government funding, administered by the Royal Society of New Zealand, and by the Carney Centre for Pharmacogenomics, University of Otago. Funding for open access charge was provided by Marsden Fund (11-UOO-175 BMS).

**REFERENCES**

Ambrus, A., D. Chen, J. Dai, R.A. Jones, and D. Yang, 2005 Solution structure of the biologically relevant G-quadruplex element in the human c-MYC promoter. Implications for G-quadruplex stabilization. *Biochemistry* 44 (6):2048-2058.

Askree, S.H., L.N. Hjelm, M.A. Pervaiz, M. Adam, L.J.H. Bean *et al.*, 2011 Allelic Dropout Can Cause False-Positive Results for Prader-Willi and Angelman Syndrome Testing. *Journal of Molecular Diagnostics* 13 (1):108-112.

Bevan, I., R. Rapley, and M. Walker, 1992 Sequencing of PCR-amplified DNA. *Genome Research* 1 (4):222-228.

Biffi, G., D. Tannahill, and S. Balasubramanian, 2012 An intramolecular G-quadruplex structure is required for TERRA RNA binding to the telomeric protein TRF2. *Journal of the American Chemical Society*.

Boán, F., M.G. Blanco, P. Barros, A.I. González, and J. Gómez-Márquez, 2004 Inhibition of DNA synthesis by K +-stabilised G-quadruplex promotes allelic preferential amplification. *FEBS Letters* 571 (1-3):112-118.

Chambers, V.S., G. Marsico, J.M. Boutell, M. Di Antonio, G.P. Smith *et al.*, 2015 High-throughput sequencing of DNA G-quadruplex structures in the human genome. *Nature biotechnology* 33 (8):877-881.

Choufani, S., J.S. Shapiro, M. Susiarjo, D.T. Butcher, D. Grafodatskaya *et al.*, 2011 A novel approach identifies new differentially methylated regions (DMRs) associated with imprinted genes. *Genome Research* 21 (3):465-476.

Desforges, J.F., and B.I. Eisenstein, 1990 The polymerase chain reaction: a new method of using molecular genetics for medical diagnosis. *New England Journal of Medicine* 322 (3):178-183.

Jirtle, J., 2012 Geneimprint Database, <http://www.geneimprint.com/>.

Lam, C.-w., and C.M. Mak, 2013 Allele dropout caused by a non-primer-site SNV affecting PCR amplification - A call for next-generation primer design algorithm. *Clinica Chimica Acta* 421:208-212.

Landsverk, M.L., G.V. Douglas, S. Tang, V.W. Zhang, G.L. Wang *et al.*, 2012 Diagnostic approaches to apparent homozygosity. *Genetics in Medicine* 14 (10):877-882.

Lek, M., K. Karczewski, E. Minikel, K. Samocha, E. Banks *et al.*, 2016 Analysis of protein-coding genetic variation in 60,706 humans. *BioRxiv*:030338.

Maizels, N., 2015 G4‐associated human diseases. *EMBO reports*:e201540607.

Morison, I.M., C.J. Paton, and S.D. Cleverley, 2001 The imprinted gene and parent-of-origin effect database. *Nucleic Acids Research* 29 (1):275-276.

Mullis, K.B., and F.A. Faloona, 1987 Specific synthesis of DNA in vitro via a polymerase-catalyzed chain reaction. *Methods in enzymology* 155:335.

Neidle, S., 2009 The structures of quadruplex nucleic acids and their drug complexes. *Current opinion in structural biology* 19 (3):239-250.

Oleg Kikin, L.D.A.a.P.S.B., 2006 QGRS Mapper: a web-based server for predicting G-quadruplexes in nucleotide sequences. *Nucleic Acids Research* 34 ((Web Server issue):W676-W682. ).

Piyamongkol, W., M.G. Bermudez, J.C. Harper, and D. Wells, 2003 Detailed investigation of factors influencing amplification efficiency and allele drop-out in single cell PCR: implications for preimplantation genetic diagnosis. *Molecular Human Reproduction* 9 (7):411-420.

Rhodes, D., and H.J. Lipps, 2015 G-quadruplexes and their regulatory roles in biology. *Nucleic Acids Research*:8627-8637.

Saiki, R.K., D.H. Gelfand, S. Stoffel, S.J. Scharf, R. Higuchi *et al.*, 1988 Primer-directed enzymatic amplification of DNA with a thermostable DNA polymerase. *Science* 239 (4839):487-491.

Saunders, C.J., M.J. Friez, M. Patterson, M. Nzabi, W. Zhao *et al.*, 2010a Allele drop-out in the MECP2 gene due to G-quadruplex and i-motif sequences when using polymerase chain reaction-based diagnosis for Rett syndrome. *Genetic testing and molecular biomarkers* 14 (2):241-247.

Saunders, C.J., M.J. Friez, M. Patterson, M. Nzabi, W.W. Zhao *et al.*, 2010b Allele Drop-Out in the MECP2 Gene Due to G-Quadruplex and i-Motif Sequences When Using Polymerase Chain Reaction-Based Diagnosis for Rett Syndrome. *Genetic testing and molecular biomarkers* 14 (2):241-247.

Sen, D., and W. Gilbert, 1988 Formation of parallel 4-stranded complexes by guanine-rich motifs in DNA and its implications for meiosis. *Nature* 334 (6180):364-366.

Simonsson, T., 2001 G-quadruplex DNA structures variations on a theme. *Biological chemistry* 382 (4):621-628.

Simsek, M., and H. Adnan, 2000 Effect of single mismatches at 3′–end of primers on polymerase chain reaction. *Journal for scientific research. Medical sciences/Sultan Qaboos University* 2 (1):11.

Stevens, A.J., S. Stuffrein-Roberts, S.L. Cree, A. Gibb, A.L. Miller *et al.*, 2014 G-Quadruplex Structures and CpG Methylation Cause Drop-Out of the Maternal Allele in Polymerase Chain Reaction Amplification of the Imprinted MEST Gene Promoter. *PLoS ONE* 9 (12):1-24.

Sun, D., and L.H. Hurley, 2010 Biochemical techniques for the characterization of G-quadruplex structures: EMSA, DMS footprinting, and DNA polymerase stop assay. *Methods in molecular biology (Clifton, N.J.)* 608:65-79.

Sundquist, W.I., and A. Klug, 1989 Telomeric DNA dimerizes by formation of guanine tetrads between hairpin loops.

Takahama, K., C. Sugimoto, S. Arai, R. Kurokawa, and T. Oyoshi, 2011 Loop Lengths of G-Quadruplex Structures Affect the G-Quadruplex DNA Binding Selectivity of the RGG Motif in Ewing’s Sarcoma. *Biochemistry* 50 (23):5369-5378.

Tomaz, R.A., B.M. Cavaco, and V. Leite, 2010 Differential methylation as a cause of allele dropout at the imprinted GNAS locus. *Genetic testing and molecular biomarkers* 14 (4):455-460.

Weitzmann, M.N., K.J. Woodford, and K. Usdin, 1996 The development and use of a DNA polymerase arrest assay for the evaluation of parameters affecting intrastrand tetraplex formation. *Journal of Biological Chemistry* 271 (34):20958-20964.

Wenzel, J.J., H. Rossmann, C. Fottner, S. Neuwirth, C. Neukirch *et al.*, 2009 Identification and Prevention of Genotyping Errors Caused by G-Quadruplex- and i-Motif-Like Sequences. *Clinical chemistry* 55 (7):1361-1371.

Yang, D., and L.H. Hurley, 2006 Structure of the biologically relevant G-quadruplex in the c-MYC promoter. *Nucleosides, Nucleotides, and Nucleic Acids* 25 (8):951-968.

Yuen, R.K., R. Jiang, M.S. Peñaherrera, D.E. McFadden, and W.P. Robinson, 2011 Genome-wide mapping of imprinted differentially methylated regions by DNA methylation profiling of human placentas from triploidies. *Epigenetics & Chromatin* 4 (1):1.

**FIGURE LEGENDS**

**Figure 1. Synthetic template ADO assay (**A) Two templates (black bars) were generated by targeted PCR amplification of genomic DNA (grey). These assay templates differed by a single basepair, which was introduced into one template (right) through primer (small green arrows) directed mutagenesis (enlarged view). (B.) An aliquot of each template was then methylated (blue circles) with M. SssI methyltransferase, and reciprocal mixing experiments were performed on each template combination as shown. In total, three template mixing experiments were performed per amplicon, for example: (1) methylated G vs non-methylated A; (2) methylated A vs non-methylated G; (3) non-methylated A vs non-methylated G.

**Figure 2. Sanger sequencing of PCR amplicons from synthetic template experiments.** Sanger sequencing analysis of eight synthetic templates derived from imprinted gene regions. Black box indicates the position of the introduced artificial SNP. (A) mix of methylated and non-methylated templates; (B) reciprocal methylated and non-methylated template mixing experiment; (C) mix of two non-methylated templates.

**Figure 3. ADO analysis of genomic *PLAGL1*.** Sanger sequencing results from PCR amplification of PLAGL1 amplicon (hg19, chr6:144328968-144328978) using primer PLAGL1Fa and PLAGL1Ra (Table S1). Black box indicates the position of SNP rs2281476. Genotyping result obtained from PCR amplification in buffer lacking KCl for DNA sample NA20588 (A) and DNA NA19312 (B). Genotyping result obtained from PCR amplification of in buffer containing 50 mM KCl for DNA sample NA20588 (C) and DNA sample NA19312 (D).

**Figure 4. Methylation dependent restriction digest performed on genomic *PLAGL1* DNA prior to PCR.**  Genotyping result obtained from PCR amplification of DNA sample NA20588 after treatment with HpaII (A) or McrBC (B). Genotyping result obtained from PCR amplification of DNA sample NA19312 after treatment with HpaII (C) or McrBC (D).

**Figure 5. CD spectra of G4 oligonucleotides at 95^o^C.** Molar ellipticity (x10^5^ deg.cm^2^.dmol^-1^) is on the vertical axis and wavelength (nm) is on the horizontal axis. Solid lines represent CD spectra in the presence of 10 mM Tris-HCl 50 mM KCl and 1.5 mM MgCl_2_, and the dashed lines represent CD spectra in 10 mM Tris-HCl and 1.5 mM MgCl_2_.
